# K_Ca_ channel blockers increase effectiveness of the EGF receptor TK inhibitor erlotinib in non-small cell lung cancer cells (A549)

**DOI:** 10.1038/s41598-021-97406-0

**Published:** 2021-09-15

**Authors:** Felix Glaser, Petra Hundehege, Etmar Bulk, Luca Matteo Todesca, Sandra Schimmelpfennig, Elke Nass, Thomas Budde, Sven G. Meuth, Albrecht Schwab

**Affiliations:** 1 Institut für Physiologie II, Westfälische Wilhelms-Universität, Robert-Koch-Str. 27b, 48149 Münster, Germany; 2 Present Address: Department of Neurology with Institute of Translational Neurology, Westfälische Wilhelms-Universität, Münster, Germany; 3 Institut für Physiologie I, Westfälische Wilhelms-Universität, Münster, Germany; 4 Present Address: Klinik für Neurologie, Universitätsklinikum Düsseldorf, Düsseldorf, Germany

**Keywords:** Physiology, Cancer, Cancer therapy, Lung cancer

## Abstract

Non-small cell lung cancer (NSCLC) has a poor prognosis with a 5 year survival rate of only ~ 10%. Important driver mutations underlying NSCLC affect the epidermal growth factor receptor (EGFR) causing the constitutive activation of its tyrosine kinase domain. There are efficient EGFR tyrosine kinase inhibitors (TKIs), but patients develop inevitably a resistance against these drugs. On the other hand, K_Ca_3.1 channels contribute to NSCLC progression so that elevated K_Ca_3.1 expression is a strong predictor of poor NSCLC patient prognosis. The present study tests whether blocking K_Ca_3.1 channels increases the sensitivity of NSCLC cells towards the EGFR TKI erlotinib and overcomes drug resistance. mRNA expression of K_Ca_3.1 channels in erlotinib-sensitive and -resistant NSCLC cells was analysed in datasets from Gene expression omnibus (GEO) and ArrayExpress. We assessed proliferation and migration of NSCLC cells. These (live cell-imaging) experiments were complemented by patch clamp experiments and Western blot analyses. We identified three out of four datasets comparing erlotinib-sensitive and -resistant NSCLC cells which revealed an altered expression of K_Ca_3.1 mRNA in erlotinib-resistant NSCLC cells. Therefore, we evaluated the combined effect of erlotinib and the K_Ca_3.1 channel inhibition with sencapoc. Erlotinib elicits a dose-dependent inhibition of migration and proliferation of NSCLC cells. The simultaneous application of the K_Ca_3.1 channel blocker senicapoc increases the sensitivity towards a low dose of erlotinib (300 nmol/L) which by itself has no effect on migration and proliferation. Partial erlotinib resistance can be overcome by K_Ca_3.1 channel blockade. The sensitivity towards erlotinib as well as the potentiating effect of K_Ca_3.1 blockade is further increased by mimicking hypoxia. Our results suggest that K_Ca_3.1 channel blockade may constitute a therapeutic concept for treating NSCLC and overcome EGFR TKI resistance. We propose that this is due to complementary mechanisms of action of both blockers.

## Introduction

Non-small cell lung cancer (NSCLC) accounts for the majority of all lung cancers^1^. The prognosis of lung cancer patients is still rather poor. Even after complete resection of early stage NSCLC ~ 50% of the patients will die from recurring tumors, and the mean 5 year survival rate of European lung cancer patients is only 13%^2^. It has become clear, that an efficient therapy needs to take into account not only the pathological classification^1^ but also the presence of genetic alterations^3, 4^. In this context, mutations of the epidermal growth factor receptor (EGFR) are of great interest. They are found in 15–50% of the non-squamous tumors. Most mutations occur in the tyrosine kinase domain of the EGFR causing its constitutive activation^5^. The knowledge of the presence or absence of EGFR mutations has great therapeutic implications since it determines the sensitivity towards blockers of the EGFR tyrosine kinase domain like erlotinib. Unfortunately, almost all patients initially responding to erlotinib and related compounds eventually develop acquired resistance towards these drugs^6, 7^. Thus, there is still an unmet need for improved therapeutic concepts for the treatment of lung cancer.

We have recently shown that K_Ca_3.1 channels play an important pathophysiological role in processes underlying NSCLC progression. They are upregulated in NSCLC biopsies, and the increased expression as well as the hypomethylation of its promoter are strong predictors for poor patient prognosis^8^ (reviewed in^9^). Increased K_Ca_3.1 channel expression promotes aggressiveness of NSCLC cells by controlling their proliferation and migration. Mechanistically, this is due to the indirect impact of K_Ca_3.1 channels on the intracellular Ca^2+^ concentration. Consequently, a blocker of K_Ca_3.1 channels, senicapoc, strongly slows down tumor progression when NSCLC cells are transplanted into mice^8^. Moreover, K_Ca_3.1 channels regulate the expression of the intercellular adhesion molecule (ICAM)1 and adhesion of NSCLC cells to endothelial cells which is an important step of the metastatic cascade^10^. While these findings clearly point to a role of K_Ca_3.1 channels in NSCLC progression, they do not address the clinical problem of resistance against tyrosine kinase inhibitors (TKI).

However, there is increasing evidence that ion channels are important players in (acquired) chemo- and/or radioresistance of cancer cells^11–14^. In ovarian cancer inhibition or silencing of K_Ca_1.1 channels leads to increased cisplatin resistance^15^. In melanoma cells the K_Ca_3.1 inhibitor TRAM-34 overcomes the resistance towards the BRAF inhibitor vemurafenib^16^. In NSCLC a link between ion channels and chemoresistance has been drawn for a voltage-gated K^+^ channel (K_V_1.1;^17^).

Here we show that K_Ca_3.1 channel expression differs between erlotinib-sensitive and -resistant NSCLC cells. We present evidence that K_Ca_3.1 and K_Ca_1.1 channel inhibition with senicapoc can exert additive inhibitory effects with the TKI erlotinib. It may even partially overcome the resistance against erlotinib.

## Methods

### Bioinformatic analysis

We used the *Gene Expression Omnibus* database (GEO; http://www.ncbi.nlm.nih.gov/geo), and *ArrayExpress* transcriptomic repository (https://www.ebi.ac.uk/arrayexpress/) to compare drug sensitive and resistant cell lines^18, 19^. We searched for microarray datasets allowing the comparison of human erlotinib-sensitive NSCLC cell lines with human erlotinib-resistant NSCLC cell lines. The raw data of four datasets (GSE31625, GSE38310, GSE38404 and GSE80344) were processed using the Bioconductor package in R (Gentleman et al. 2004). They were background corrected and then normalized with a log_2_ conversion. A matrix was created for each dataset after the processing optimization, in order to compare expression data from erlotinib-sensitive and erlotinib-resistant NSCLC cell lines. The analysis of the matrix was carried out with a linear model (limma software in R), in order to identify the differentially expressed genes in the two groups^20^. An impartial cut-off of 0.05 was imposed on the section for identifying the differentially expressed genes between the two groups.

### Cell culture

A549 lung adenocarcinoma cells were cultured at 37 °C in a 5% CO_2_ atmosphere in Dulbecco’s modified Eagle’s medium (Invitrogen, Carlsbad, CA) supplemented with 10% fetal calf serum. The generation of the highly aggressive A549 cell line, designated as A549-3R, was described previously^21^. H1975 cells were cultured in RPMI1640 medium supplemented with L-glutamine and 10% FCS. Hypoxia was produced by incubating the A549 cells in 5% CO_2_ and 0.2% O_2_. To obtain A549-3R cells that are partially resistant against erlotinib they were cultured in the presence of 10 µmol/L erlotinib (Chemos GmbH, Germany) and 10 ng/mL EGF (Sigma Aldrich) for nine weeks. Thereby, we wanted to mimick the situation encoutered in patients whose serum levels were determined to be ~ 2 ng/ml^22^. After thawing cryoconserved erlotinib-resistant A549-3R cells they were cultured for two weeks in the presence of erlotinib and EGF prior to use for experiments.

### Proliferation

50.000 cells each were seeded in 12-well plates in triplicate (Falcon Multiwell 12 Well Tissue Culture Plate, Becton Dickinson, NJ, USA). After 24 h growth in standard cell culture medium, cells were starved in serum-free medium for another 24 h. Thereafter, experimental media supplemented with 10% FCS, 100 ng/mL EGF and drugs (erlotinib, senicapoc, DMSO, dimethyl-oxaloyl-glycine (DMOG; 1 mmol/l) as indicated) were added. Experimental media were changed every other 24 h. Cells were counted in a Neubauer chamber after serum starvation (t = 0) and following trypsinization after 24, 48 and 72 h, respectively.

### Cell migration

Cell migration experiments were performed as described previously^8^. Briefly, A549-3R cells were seeded on matrices of the following composition: RPMI (10.4 g/L), HEPES (10 mmol/L), laminin (20 µg/mL; Sigma Aldrich), fibronectin (40 µg/µL; Becton Dickinson, USA), collagen IV (5.4 µg/µL; Becton Dickinson), collagen III (12 µg/µL; Becton Dickinson), collagen I (800 µg/mL; Biochrom, Germany), H_2_O ad 1000 µL, titrated to pH 7.4 with 1 N NaOH. We used ~ 200 µL of the matrix per 12.5 cm^2^ culture flask. The next day A549-3R cells were seeded onto the matrix. They adhered to the matrix for 4 h prior to the start of the experiment. The culture medium used for the migration experiments was supplemented with 100 ng/mL EGF and different concentrations of senicapoc and/or erlotinib. The solvent, DMSO, was added in equal amounts (1:1000) for control experiments. Images were acquired in 10 min intervals for a total duration of 10 h and migration was quantified from the movement of the cell center as described previously^23^. Migration speed is calculated as a three point difference quotient for each time interval, translocation refers to the net movement during the course of the experiment, i. e. the distance between the start and end position of the cell. The directionality is determined by dividing the translocation by the total path length covered by the cell during the course of the experiment. Thus, directionality can be seen as a measure of the tortuosity of the cell path.

### Single-cell electrophysiology

Whole-cell patch clamp recordings were performed at room temperature using borosilicate glass pipettes (GC150TF-10, Clark Electromedical Instruments, Pangbourne, UK) connected to an EPC-10 amplifier (HEKA Electronics, Lambrecht, Germany) as described previously^24, 25^. The typical electrode resistance was 4–5 MΩ, while series resistance was in the range of 8–15 MΩ. Series resistance compensation of > 30% was used. Voltage clamp experiments on cultured cells were controlled by Patch Master software (HEKA Electronics, Lambrecht, Germany). Current density was calculated by dividing the current amplitude determined at the end of the depolarizing voltage ramp to + 60 mV by the membrane capacitance obtained from slow capacitance compensation. The following recording solutions were used: (a) extracellular solution (in millimoles per liter): NaCl, 140; KCl, 5; HEPES, 10; MgCl_2_, 1; CaCl_2_, 1; pH 7.4 with NaOH. (b) Intracellular solution (in millimoles per liter): KCl, 140; HEPES, 10; EGTA, 1.3; CaCl_2_, 1.217; MgCl_2_,1; pH 7.4 with KOH. The calculated free Ca^2+^ concentration of the internal solution was set to 1 μmol/l in order to obtain full activation of the K_Ca_ channels during the patch clamp experiments. A liquid junction potential of − 3.2 mV was not taken into account. Drugs were dissolved in DMSO and added to the standard extracellular solution and applied for a time period of 10 min. A multi-barrel application pipette with a tip diameter of about 100 μm was used for drug application close to the recorded cell. Recordings were analyzed using Fit Master and Origin 7.5 software. First the total transmembrane current flow (sum of inward and outward currents mediated by all open channels) in response to the voltage clamp protocol was measured as control. In order to increase the current proportion mediated by members of the family of calcium-activated K^+^ channels, the activator 1-EBIO (50 µmol/L) was added to the bath solution. Next, the K_Ca_1.1 channel blocker paxilline (10 µmol/L) was applied to the bath solution containing 1-EBIO. Finally, the K_Ca_3.1 channel blocker senicapoc (1 µmol/L) was added to the cocktail. K_Ca_1.1- and K_Ca_3.1-mediated currents are represented by the paxilline-sensitive and senicapoc-sensitive current components, respectively.

### Western blotting

We employed standard techniques for Western blotting described previously^8^ and used the following antibodies: anti-K_Ca_3.1 (1:500; Sigma), anti-EGFR (1:250; Santa Cruz Inc., USA), anti-HIF-1α (1:1000; Becton Dickinson), anti-phospho-p44/42 (pERK1/2; 1:1,000; Cell Signaling Technology Inc., USA; cat. # 9106), anti-actin (1:10,000, Sigma Aldrich). When PVDF membranes were probed successively with multiple antibodies, they were stripped by incubation in 200 mmol/L NaOH for 5 min and subsequent washing.

### Statistics

Data are presented as mean ± SEM unless otherwise indicated. N reflects the number of independent experiments, n represents the number of individual cells. Extreme values (3 interquartile ranges above the third or below the first quartile) were removed and data were tested for normality. Significance (*p* < 0.05) was assessed by One-way-ANOVA or Kruskal–Wallis test followed by Bonferroni or Fisher post hoc test. Migration experiments were also evaluated with mixed-measures ANOVA and automatic post hoc test.

## Results

### Altered expression of K_Ca_3.1 channels in erlotinib-resistant NSCLC cells

**Figure 1:**
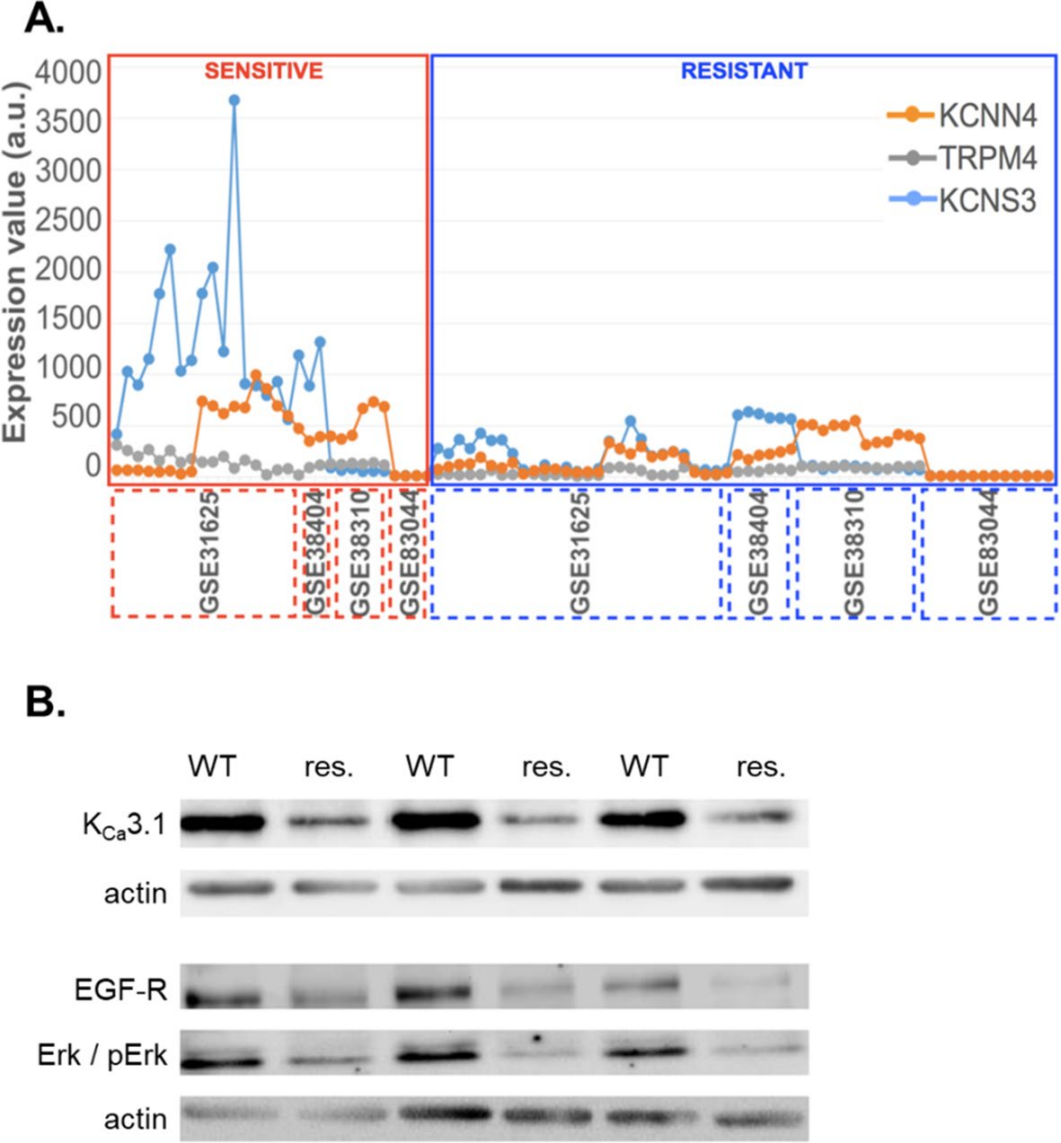
(**A**) Gene expression values (estimate of the number of RNA transcripts) of KCNN4, TRPM4 and KCNS3 for each cell line represented by the GSE31625, GSE38310, GSE80344 and GSE38404 microarray datasets. The gene expression values have been normalized by removing non-biological variation, and generating the final probe set expression values. GSE31625 gene expression was evaluated with MAS5 normalization, GSE38310, GSE80344 and GSE38404 were evaluated with RMA normalization. The x axis represents all the samples from the four datasets. (**B**) Western blot analysis of K_Ca_3.1 channels, EGFR and phospho-p44/42 (pERK1/2) in wildtype A549-3R (WT) and partially erlotinib resistant A549-3R (res.) cells. Western blots were repeated three times (N = 3).

We searched the GEO and ArrayExpress databases for mRNA expression obtained from microarrays of human erlotinib-sensitive and erlotinib-resistant cell lines and identified four datasets of interest: GSE31625, GSE38310, GSE38404 and GSE80344. They contained data from 18, 6, 2 and 4 NSCLC erlotinib-sensitive cell lines as well as 28, 12, 6, and 12 NSCLC erlotinib-resistant cell lines, respectively. On average, these datasets displayed ~ 4.100 genes that were differentially expressed in the two groups when applying a threshhold of 0.05 for the adjusted p-value. Using the online software Venny 2.0 (http://bioinfogp.cnb.csic.es/tools/venny/index.html) we found 23 differentially expressed genes that are common to all four datasets, and 504 are common at least to 3 of the 4 datasets. We identified three ion channel genes that are expressed at a reduced level in erlotinib-resistant NSCLC cell lines. *KCNN4* (encoding the K_Ca_3.1 channel) and *TRPM4* (encoding TRPM4 channels) are listed in GSE31625, GSE38310 and GSE38404 with logFC values (number of times a gene is over/under-expressed) of − 2.82, 0.41 and − 0.86 (*KCNN4*) as well as − 2.16, − 0.40 and − 0.75 (*TRPM4*), respectively. *KCNS3* which encodes the K_V_9.3 channel is detected in GSE31625, GSE80344 and GSE38404, with logFC values of − 1.21, − 1.81 and − 0.75, respectively. These results are summarized in Fig. 1A. The expression of other K_Ca_ channels such as K_Ca_1.1 does not differ between erlotinib-sensitive and -resistant cell lines in these data sets.

In the present study we followed up on K_Ca_3.1 channels and their potential functional role in erlotinib-ressitance of NSCLC cells using the A549-3R cell model that we had characterized previously^8^. To learn more about the role of EGFR signaling and K_Ca_3.1 in NSCLC cells, we generated partially erlotinib-resistant A549-3R cells by culturing them for ~ 9 weeks in the presence of 10 µmol/L erlotinib and 100 ng/ml EGF. Western bot analysis recapitulates our in silico analyses and reveals that K_Ca_3.1 expression is reduced in these cells (N = 3). Moreover, EGFR expression and ERK phosphorylation are also diminished (see Figs. 1B and 1Ba-suppl). For comparison, we also tested H1975 cells that are erlotinib-resistant because of an activating mutation of the EGFR which is then constitutively active. They are also represented in arrays mentioned above and have a lower mRNA and protein expression of K_Ca_3.1 channels than erlotinib-sensitive NSCLC cells (data not shown). K_Ca_3.1 expression is lower in H1975 cells than in wt A549-3R cells (N = 3; Fig. 1Bb-suppl.).

### Functional K_Ca_ channels in wildtype and partially erlotinib-resistant A549-3R cells

**Figure 2:**
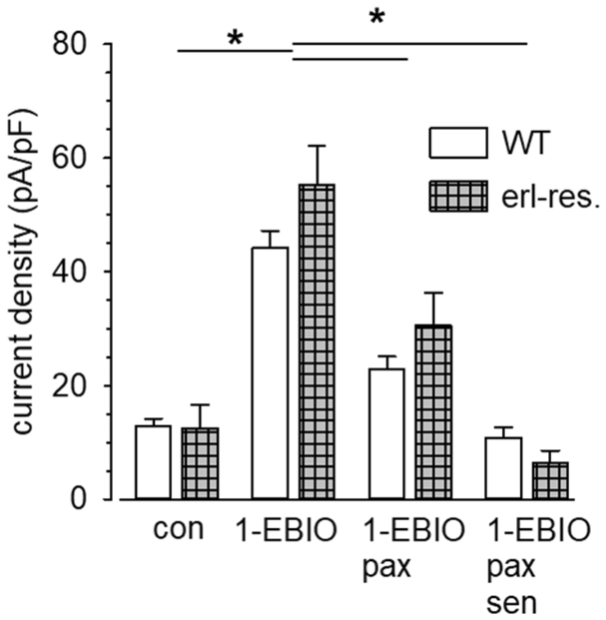
Summary of patch clamp experiments. We compared the mean current densities @ + 60 mV of erlotinib-sensitive WT A549-3R and erlotinib-resistant A549-3R cells. The contribution of K_Ca_3.1 and K_Ca_1.1 channels to the outward current induced by the application of 1-EBIO (50 µmol/L) is disclosed by adding the K_Ca_1.1 blocker paxilline (pax; 10 µmol/l) or the K_Ca_3.1 blocker senicapoc (sen; 1 µmol/L). A549-3R WT: N = 9, erlotinib-resistant cells: N = 10. * indicates *p* < 0.05.

We next tested whether the altered K_Ca_3.1 expression on mRNA and protein level also affects channel activity. We therefore performed patch clamp experiments with wildtype and partially erlotinib-resistant A549-3R cells. First we compared K_Ca_ current densities when cells were kept under standard cell culture conditions (ambient pO_2_). We had characterized K_Ca_3.1-mediated current in A549-3R cells already previously^8^. We slightly modified the protocol and also probed for K_Ca_1.1 channels (paxillin-sensitive current component) because they are known to be activated by 1-EBIO, too^26^. Moreover, we used senicapoc to inhibit K_Ca_3.1-mediated currents. Figure 2 provides a summary of the patch clamp experiments. Current density (@ + 60 mV) in A549-3R WT cells rises ~ fourfold from 12.8 ± 1.4 pA/pF to 44.1 ± 3.1 pA/pF (n = 9) following the application of 1-EBIO (50 µmol/L). The 1-EBIO-induced increase of the current density is largely due to the activation of paxilline-sensitive K_Ca_1.1 channels. Under normoxic conditions the paxilline-sensitive current density amounts to 21.2 ± 2.9 pA/pF, while the senicapoc-sensitive current component is only 12.0 ± 1.6 pA/pF.

Partially erlotinib-resistant cells respond in a similar fashion (see Fig. 2). There is a trend towards higher K_Ca_3.1 current density in partially erlotinib-resistant than in A549-3R WT cells. In these cells it mediates almost as much current as K_Ca_1.1 channel activity. However, this does not reach statistical significance when compared with A549-3R WT cells. Thus, K_Ca_3.1 channel activity behaves differently than the channel expression on mRNA and protein level. We next tested the impact of these channels on the sensitivity of A549-3R cells towards erlotinib, an EGFR TKI.

### Erlotinib inhibits migration and proliferation of A549-3R cells

**Figure 3:**
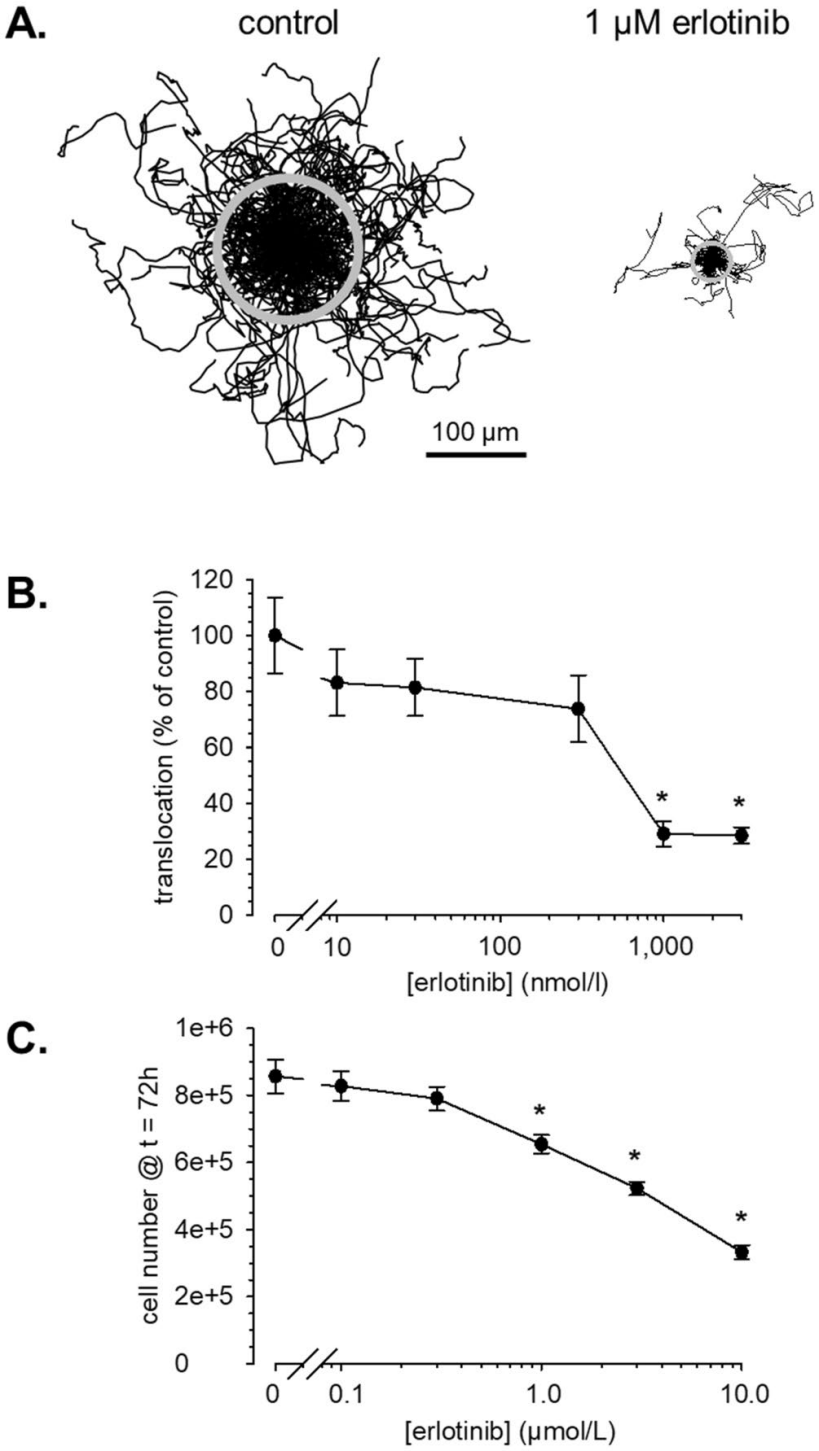
Erlotinib induces a dose-dependent inhibition of migration and proliferation of A549-3R cells. (**A**) Trajectories of migrating A549-3R cells. Each line corresponds to the path of an individual cell. Trajectories are normalized to a common starting point. The radius of the grey circle represents the translocation, i. e. the mean distance between start and end point of the cells. N/n = 14/168 for control and N/n = 5/66 for 1 µmol/L erlotinib. (**B**) Summary of migration experiments. Erlotinib reduces translocation dose-dependently. Translocation is normalized to the respective control that were always run in parallel. (**C**) Proliferation of A549-3R cells is also inhibited dose-dependently by erlotinib (N = 4). The cell number @ t = 72 h after plating is normalized to the respective control values. * indicates *p* < 0.05.

In a first set of functional experiments we quantified the inhibitory effect of erlotinib, an inhibitor of the EGFR tyrosine kinase, on migration of A549-3R cells. Erlotinib was applied in a concentration range of 10 nmol/l to 3 µmol/L. Control experiments in the presence of DMSO (1:1000) were always performed in parallel. Under control conditions migration speed is 0.40 ± 0.02 µm/min and translocation amounts to 77.9 ± 4.6 µm (n = 168 cells from N = 14 experiments). Figure 3A depicts the trajectories normalized to a common starting point for cells migrating under control conditions and in the presence of 1 µmol/L erlotinib. Erlotinib leads to a marked inhibition of migration. The dose-response curve shown in Fig. 3B indicates that the IC_50_ value is in the order of ~ 500 nmol/L. Similarly, proliferation of A549-3R cells is inhibited by erlotinib (Fig. 3C). Consistent with published results^27^ the estimated IC_50_ value is in the order of 5 µmol/L. Thus, erlotinib inhibits migration more efficiently than it prevents an increase of the cell number. Moreover, these concentrations correspond well to clinically achievable plasma concentrations in patients^28^.

Based on these results, the experiments combining erlotinib with the K_Ca_3.1 blocker senicapoc or the K_Ca_1.1 blocker paxilline will be performed either with 300 nmol/L, 3 µmol/L or 10 µmol/L erlotinib. These concentrations are not yet effective in inhibiting migration and proliferation (300 nmol/L), or they produce the maximal effects (3 µmol/L and 10 µmol/L).

### Combining erlotinib with senicapoc or paxilline elicits additive effects on migration and proliferation

**Figure 4:**
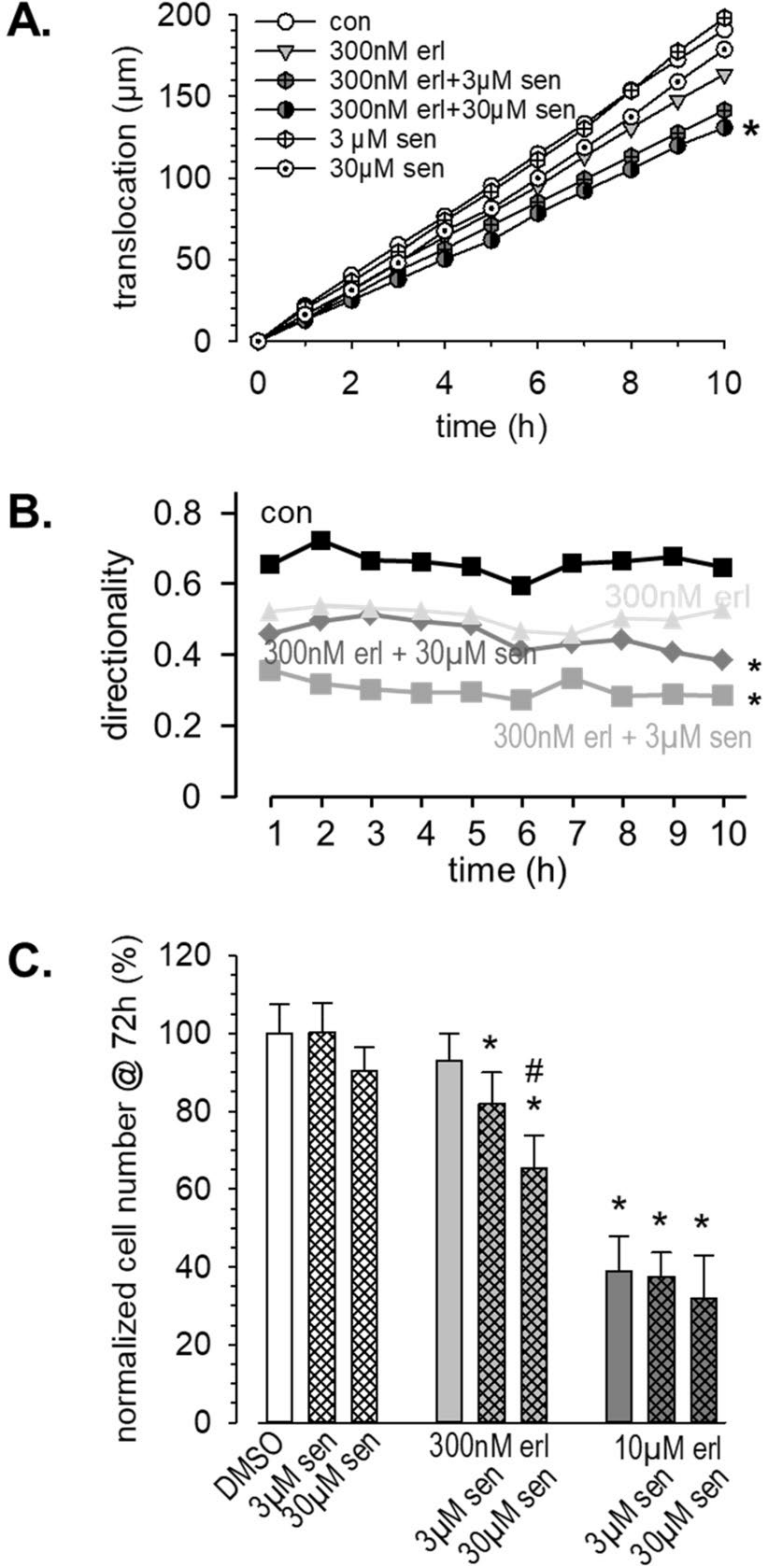
Combining erlotinib with the K_Ca_3.1 inhibitor senicapoc elicits additive effects on migration and proliferation. (**A**) Summary of migration experiments. We plotted the cumulative translocation (binned in 1 h intervals) during the course of the experiments. For clarity we omitted the error bars. (N/n ≥ 3/30). (**B**) Combining erlotinib and senicapoc leads to a reduction of the directionality of migrating cells. (**C**) Summary of proliferation experiments (N = 4). * indicates *p* < 0.05 with respect to the DMSO control; # indicates *p* < 0.05 with respect to 300 nmol/L erlotinib alone.

We combined 300 nmol/L erlotinib with 3 µmol/L or 30 µmol/L senicapoc in migration experiments. The results of these experiments are summarized in Fig. 4. The combination of both drugs reduces the translocation (Fig. 4A), while either drug alone has no significant impact on migration. The inhibition of migration by the drug combination can be accounted for by a reduction of the directionality of migration (Fig. 4B). When combining a tenfold higher concentration of erlotinib (3 µmol/L) with senicapoc, the K_Ca_3.1 channel blocker does not augment the inhibition of migration that is already elicited by erlotinib alone. The respective mean values of the translocation are (N = 3 experiments each): DMSO: 101 ± 12 µm (n = 30); 3 µmol/L erlotinib: 45 ± 8 µm (n = 30); 3 µmol/L erlotinib plus 3 µmol/L senicapoc: 45 ± 7 µm (n = 30); 3 µmol/L erlotinib plus 30 µmol/L senicapoc: 52 ± 8 µm (n = 29).

We observed a similar pattern in proliferation experiments (Fig. 4C). Combining low dose erlotinib (300 nmol/L) with the K_Ca_3.1 channel blocker senicapoc inhibits proliferation. It is reduced by 18% and 35% in the combined presence of 300 nmol/L erlotinib and 3 or 30 µmol/L senicapoc, respectively. Moreover, the cell number is significantly lower in the combined presence of 300 nmol/L erlotinib and 30 µmol/L senicapoc than in the presence of erlotinib alone. We also tested the combination of senicapoc (3 and 30 µmol/L) with 10 µmol/L erlotinib. Erlotinib alone and in combination with senicapoc reduces the cell number by 61%, 62% and 68%, respectively. Thus, K_Ca_3.1 channel inhibition has only a minor additional effect in the presence of a high dose of erlotinib.

**Figure 5:**
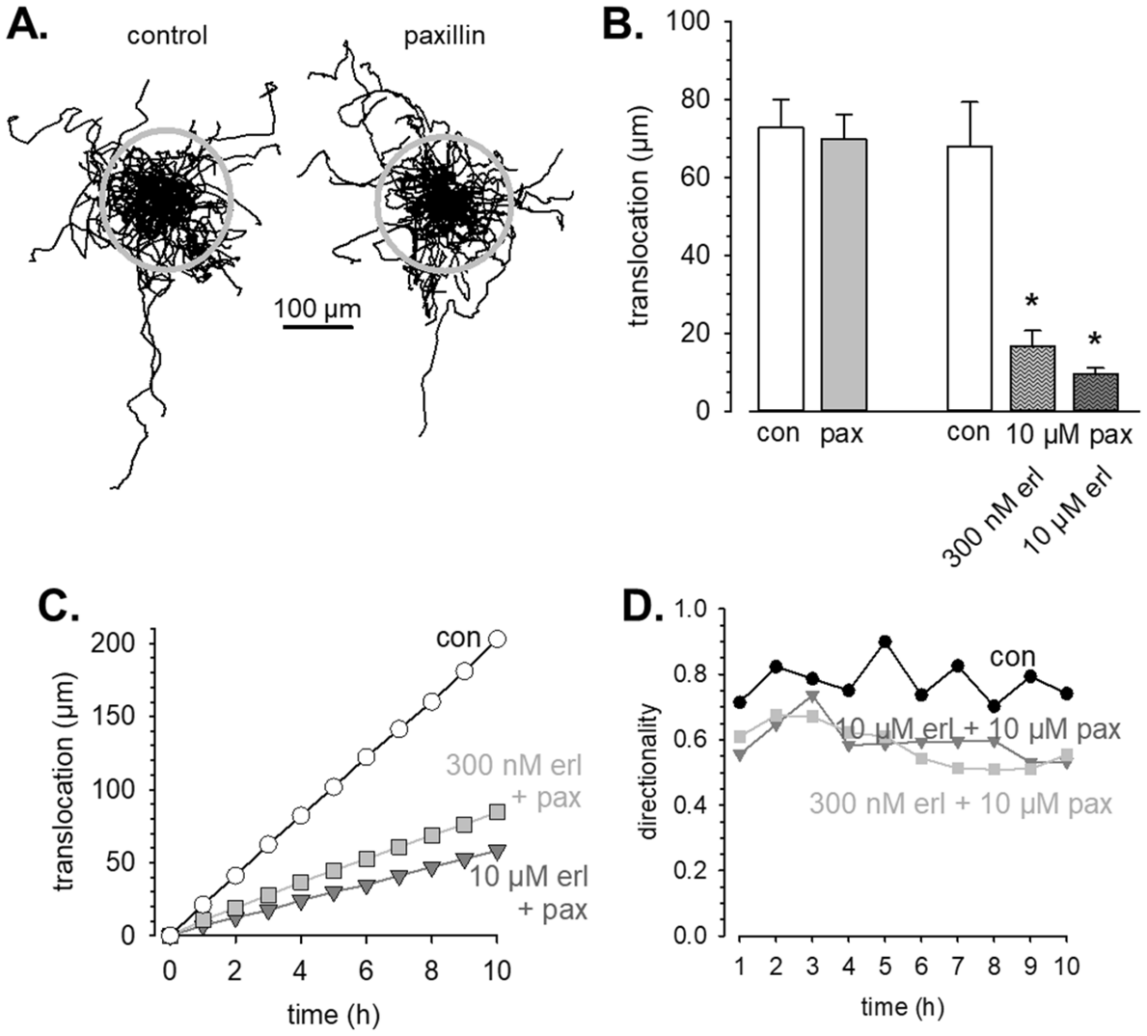
K_Ca_1.1 channel blockade potentiates the inhibitory effect of erlotinib on migration of A549-3R cells. (**A**) Trajectories of individual A549-3R cells migrating under control conditions (left panel; n = 59) or in the presence of 10 µmol/L paxilline (right panel; n = 60). Cell paths are normalized to common starting points and the grey circles represent the mean translocations covered during the course of the experiment. (**B**) Summary of the migration experiments in which we probed the contribution of K_Ca_1.1 channels with paxilline (pax; 10 µmol/L) under control conditions (con; left part of the panel) and in combination with erlotinib (erl; right part of the panel; N/n = 3/30 for each condition). (**C**) Cumulative translocation binned in 1 h intervals of A549-3R cells migrating under control conditions and in the presence of paxilline and erlotinib. (**D**) The directionality of A549-3R cells is reduced in the presence of paxilline and erlotinib.

We also tested the effect of inhibiting K_Ca_1.1 channels in A549-3R cells (Fig. 5). Figure 5A displays the trajectories of A549-3R cells migrating under control conditions and in the presence of 10 µmol/l paxilline. It is evident that K_Ca_1.1 channel inhibition by itself does not affect migration. Translocation of control and paxilline-treated cells amounts to 72.7 ± 7.3 µm (n = 59; Fig. 5A left panel and Fig. 5B) and 69.8 ± 6.4 µm (n = 60; Fig. 5A, right panel and Fig. 5B), respectively. However, paxilline and erlotinib elicit strongly potentiated inhibitory effects when they are combined. The additional application of the low dose of erlotinib (300 nmol/l) to 10 µmol/l paxilline reduces the translocation by ~ 75% to 16.8 ± 3.9 µm. Combining 10 µmol/l paxilline with 10 µmol/l erlotinib causes a reduction of the translocation by ~ 85% to 9.6 ± 1.5 µm (n = 30 each; Fig. 5B). We plotted the cumulative translocation (covered during 1 h intervals) as a function of time in Fig. 5C. It becomes evident that the inhibition of migration is effective during the entire course of the experiment. In addition to the speed of migration, the combination of erlotinib and paxilline also reduces the directionality by ~ 25% (Fig. 5D).

### Partial erlotinib resistance can be overcome by K_Ca_3.1 or K_Ca_1.1 channel blockade

**Figure 6:**
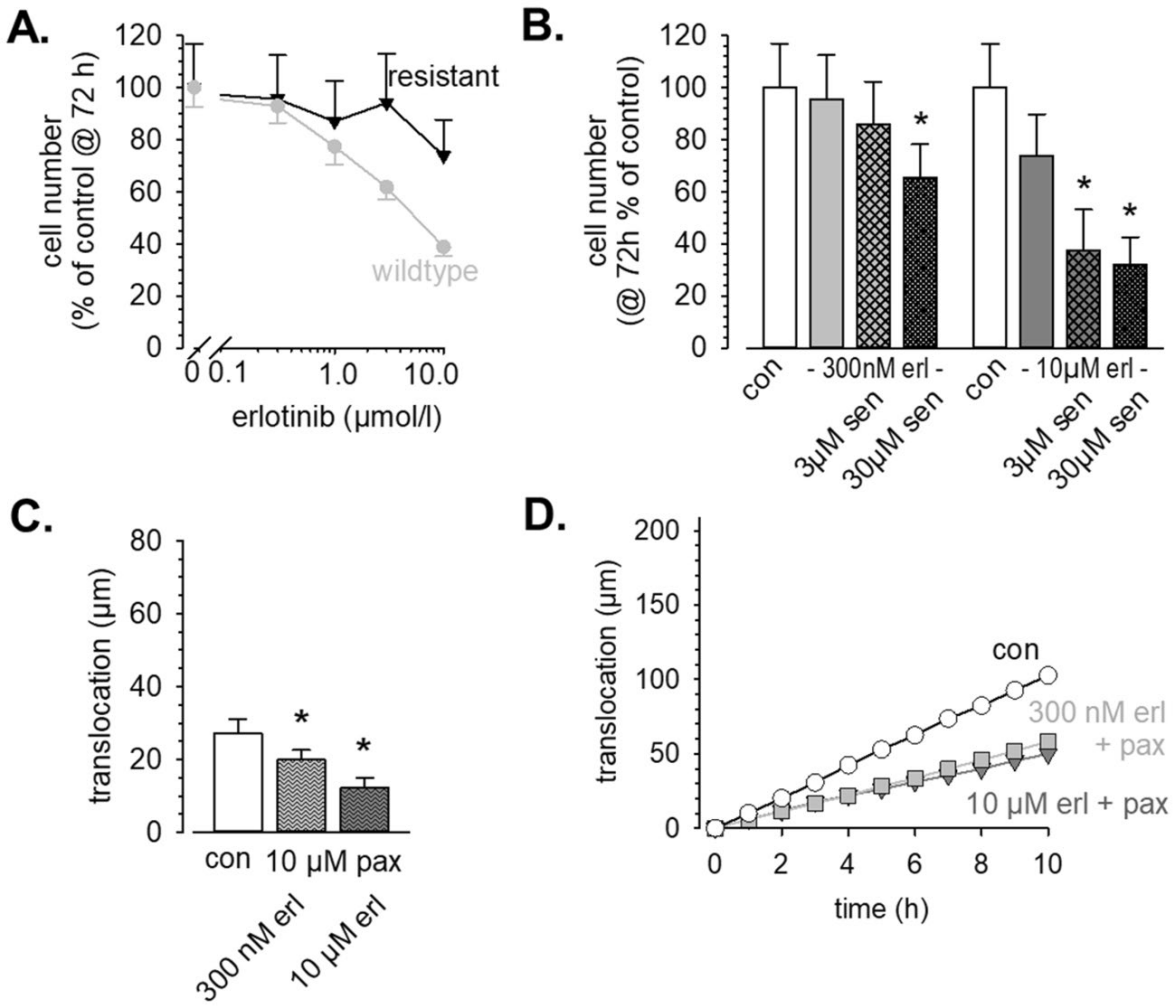
K_Ca_3.1 and K_Ca_1.1 inhibition can overcome partial erlotinib resistance. (**A**) Dose–response curves of proliferation for erlotinib. For comparison, we replotted data from Fig. 1A for wildtype cells (grey symbols). (**B**) Proliferation of partially erlotinib resistant A549-3R cells is efficiently inhibited by the K_Ca_3.1 channel blocker senicapoc. (**C**, **D**) Summary of migration experiments with partially erlotinib-resistant A549-3R cells (N/n = 3/30). Blocking K_Ca_1.1 channels with paxillin causes a marked inhibition of migration.

As expected, proliferation with erotinib-resistant cells experiments revealed a ~ tenfold lower sensitivity towards EGFR tyrosine kinase inhibition with erlotinib (Fig. 6A). 10 µmol/L erlotinib only cause a reduction of the cell number @ t = 72 h by 27%. For comparison, 1 µmol/L erlotinib inhibits proliferation by 24% in control cells. We then combined erlotinib with the K_Ca_3.1 channel blocker senicapoc. When using 300 nmol/L erlotinib which by itself is ineffective (− 6%) together with 3 µmol/L or 30 µmol/L senicapoc, proliferation is reduced by 15% or 35%, respectively. When using 10 µmol/L erlotinib together with 3 µmol/L or 30 µmol/L senicapoc, proliferation is reduced by up to ~ 70% (Fig. 6B). Thus, in partially erlotinib-resistant cells K_Ca_3.1 channel blockade elicits a more pronounced effect than in control cells and thereby partially overcomes erlotinib resistance.

The K_Ca_1.1 channel blocker paxilline (10 µmol/l) produces comparable results in migration experiments with partially erlotinib-resistant A549-3R cells. It is notable that the translocation of these cells is strongly reduced when compared with the parental WT cells. They cover only 27.2 ± 4.0 µm during the course of the experiments compared to 67.8 ± 11.5 µm. In the combined presence of paxilline and erlotinib the translocation of the partially erlotinib-resistant cells is reduced by further ~ 50% (N/n = 3/30 each; Fig. 6C,D).

### Hypoxia augments the contribution of K_Ca_3.1 channels to migration and proliferation

Finally, we assessed the impact of K_Ca_ channel activity on NSCLC cells when they were kept under hypoxic conditions. We included the analysis of hypoxia because it has been known for many years that the NSCLC tumor stroma is hypoxic^29^. Moreover, it has been shown that K_Ca_3.1 channels are upregulated in melanoma cells when they are cultured under hypoxic conditions^30^.

**Figure 7:**
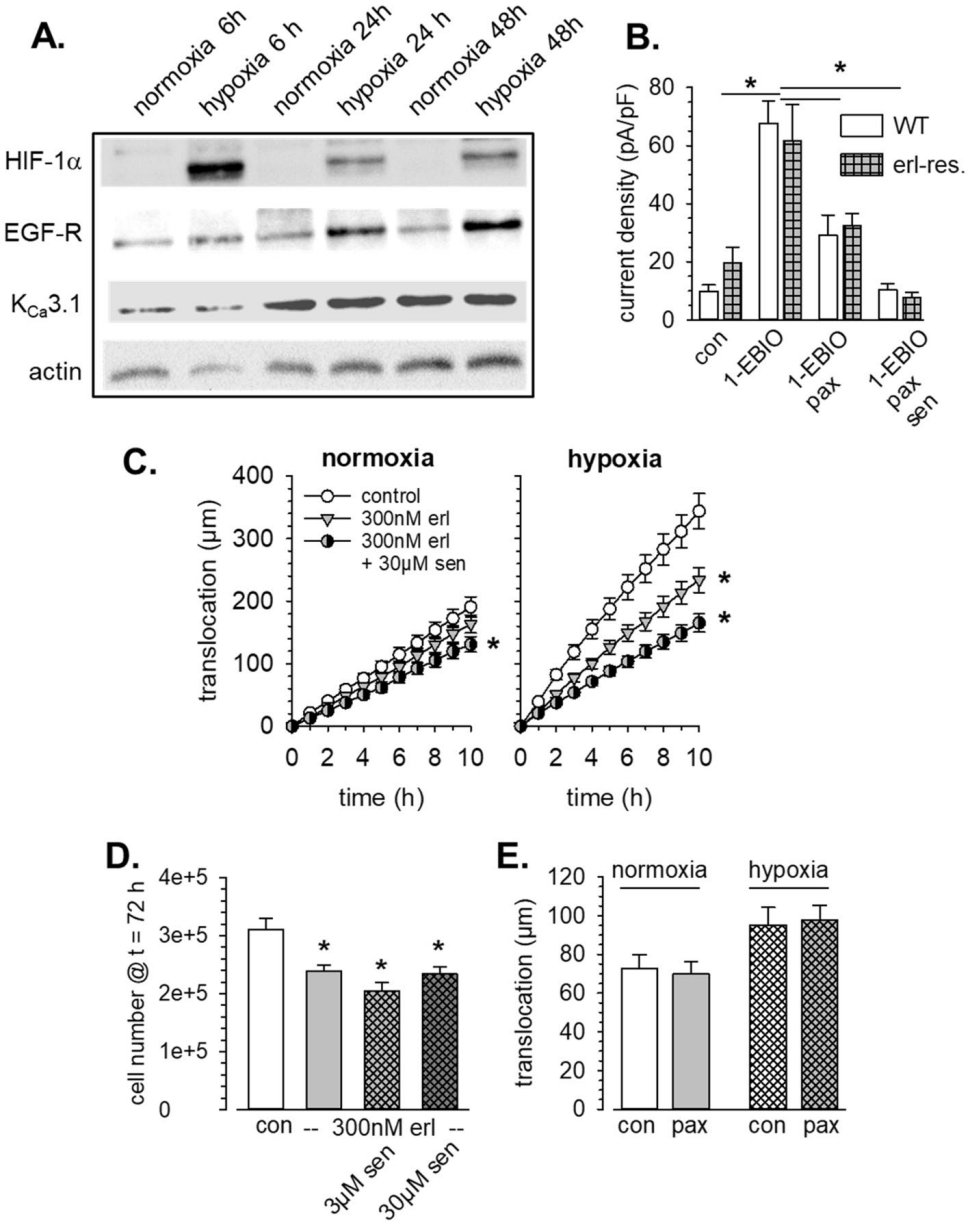
Hypoxia sensitizes A549-3R cells for EGFR and K_Ca_3.1 channel inhibition. (**A**) Western blot analysis reveals increased expression of HIF-1α and EGFR while that of K_Ca_3.1 channels is not altered. The blots are representative for N = 3 experiments. (**B**) Current densities of erlotinib-sensitive WT A549-3R (N = 12) and erlotinib-resistant A549-3R cells (N = 8). **C.** Comparison of the cumulative translocation under normoxia and under hypoxia. In the presence of chemical hypoxia both low dose erlotinib (300 nmol/L) and K_Ca_3.1 inhibition reduce migration more effectively than under normoxic conditions (N/n = 3/30). (**D**) Summary of proliferation experiments in the presence of chemical hypoxia. Note that the proliferation rate is only ~ 1/3 of that in normoxic conditions. *Indicates *p* < 0.05 with respect to the DMSO control. (**E**) Hypoxia does not increase the sensitivity of A549-3R cells towards inhibition of K_Ca_1.1 channels. For comparison, we replotted the data for normoxia from Fig. 5B.

We pretreated A549-3R cells with 1 mmol/L DMOG or kept them under hypoxic conditions (0.2% O_2_) for up to 72 h. Six hours of hypoxia lead to a massively elevated expression of HIF-1α in A549-3R (Fig. 7A). Thereafter, HIF-1α decreases but still remains on a higher level than in control cells. The EGFR expression is also strongly increased and remains steadily on a higher level. In contrast, K_Ca_3.1 expression itself is not altered by hypoxia which is in contrast to results obtained from melanoma cells^30^. Similarly, K_Ca_1.1 channels were shown to be activated in glioblastoma cells in response to hypoxia^31^. In order to prevent a transient reoxygenation upon addition of inhibitors during migration and proliferation experiments, we induced a chemical hypoxia during the functional assays. Although the numerical values of the observed changes of current densities are bigger in hypoxia-adapted cells, they do not reach statistical significance when compared with the values obtained under normoxia. Under hypoxic condition current density rises almost sevenfold, from 9.8 ± 2.2 pA/pF to 67.7 ± 7.7 pA/pF (n = 12). The K_Ca_1.1-mediated K^+^ current density doubles and rises to 38.6 ± 9.8 pA/pF. K_Ca_3.1-mediated current increases by 64% and reaches 18.7 ± 6.8 pA/pF (see Fig. 7B). Thus, our patch clamp experiments confirm the Western blot results in that K_Ca_3.1 channel activity, blocked by senicapoc (1 µmol/L), is not massively increased by hypoxia. WT cells (erlotinib-sensitive) and erlotinib and erlotinib-resistant cells behave alike in this respect.

It is notable that DMOG-pretreated A549-3R cells migrate considerably faster than control cells. Speed of migration and translocation amount to 0.51 ± 0.05 µm/min and 102 ± 15 µm, respectively. Furthermore, cells are sensitized towards erlotinib (300 nmol/L) in the presence of chemical hypoxia: Migration is reduced more efficiently than under normoxic conditions (0.31 ± 0.03 µm/min and 56 ± 9 µm corresponding to a reduction of ~ 40% ). The same holds true for the additional effect of K_Ca_3.1 channel blockade. The respective values are 0.25 ± 0.03 µm/min and 50 ± 11 µm for 300 nmol/L erlotinib plus 3 µmol/L senicapoc and 0.21 ± 0.03 µm/min and 47 ± 14 µm for 300 nmol/L erlotinib plus 30 µmol/L senicapoc which correspond to reductions by ~ 50% and ~ 60%, respectively (N/n = 3/30; *p* < 0.05). This effect of the K_Ca_3.1 channel blocker senicapoc is additive when cells are treated with 3 µmol/L erlotinib (data not shown). The increased efficacy of the combination of low concentrations of erlotinib and senicapoc to inhbit migration of A549-3R cells under hypoxic conditions becomes particularly evident when plotting the cumulative translocation (see Fig. 7C).

Proliferation of A549-3R cells is much slower in the presence of chemical hypoxia (Fig. 7D). Yet, proliferation is further reduced by erlotinib and by its combination with the K_Ca_3.1 channel blocker senicapoc. Notably, the lowest (combined) concentrations of erlotinib and senicapoc already elicit significant effects and reduce the cell number by 23% (300 nmol/l erlotinib) or 34% (300 nmol/l erlotinib plus 3 µmol/l senicapoc; Fig. 7D; N = 4). Taken together, the combined application of EGFR tyrosine kinase and K_Ca_3.1 channel blockade leads to an effective inhibition of migration in the presence of chemical hypoxia.

In contrast, chemical hypoxia does not increase the sensitivity of A549 cells to K_Ca_1.1 channel blockade. Paxilline (10 µmol/l) has no effect on the migration of A549-3R cells pretreated with DMOG (Fig. 7E). For cells kept under hypoxia, the respective values are 95.2 ± 9.2 µm (n = 55) and 97.6 ± 7.8 µm (n = 53). This underlines the distinct roles played by K_Ca_3.1 and K_Ca_1.1 channels in regulating NSCLC cell behavior.

## Discussion

Our study addressed the question whether the therapeutic efficacy of the EGFR tyrosine kinase inhibitor erlotinib can be enhanced and therapy resistance be overcome by combining it with K_Ca_ channel blockers. Since TKI resistance is a particular problem in NSCLC we performed our experiments with the aggressive lung adenocarcinoma cell line A549-3R. Our key results are the following: (i) Depending on the concentration of erlotinib, the simultaneous application of the K_Ca_3.1 inhibitor senicapoc or the K_Ca_1.1 blocker paxilline elicit additive effects on the reduction of proliferation and cell migration. (ii) Hypoxia and partial erlotinib resistance increase the susceptibility to K_Ca_3.1 channel inhibition while this appears not to be the case for K_Ca_1.1 blockade. Thus, K_Ca_ channel inhibition has the potential to strengthen the therapeutic efficacy of drugs such as erlotinib and to potentially overcome EGFR TKI resistance.

At first sight, the senicapoc concentrations used in the migration and proliferation experiments appear to be exceedingly high. The nominal concentrations are much higher than those used in patch clamp experiments. However, it has to be kept in mind that K_Ca_3.1 channel inhibitors having a similar structure as the lead compound clotrimazole, are strongly bound by plasma proteins so that the free concentration is up to 50 times lower^32^. In this submicromolar concentration range senicapoc is a highly specific inhibitor of K_Ca_3.1^33^. Moreover, it is conceivable that the observed effects may at least be in part be mediated by mitochondrial K_Ca_3.1 channels (see discussion below). Then the blockers have to cross the plasma membrane and the outer mitochondrial membrane to reach the K_Ca_3.1 channel protein in the inner mitochondrial membrane. Obviously, effective drug concentrations are not easy to predict at this location.

The additive effects of EGFR TKIs and K_Ca_3.1 and K_Ca_1.1 channel blockers can be explained by considering the differential mechanisms by which both membrane proteins regulate cell proliferation and migration. EGFR activates several signaling pathways such as RAS/RAF/MAPK, PI3K/AKT and JAK/STAT thereby leading to progression of the cell cycle, proliferation and an inhibition of apoptosis^34^. Inhibition of these signaling cascades causes a cell cycle arrest in G_1_/G_0_^35^. Moreover, there is evidence that erlotinib promotes apoptosis by elevating the intracellular ROS production^36^. K_Ca_3.1 inhibition has complementary and synergistic effects. It arrests the cell cycle at the G_1_/S phase transition^37, 38^. K_Ca_3.1 and K_Ca_1.1 channels are also expressed in the inner mitochondrial membrane of cancer cells^39, 40^. Blocking the channels is expected to elicit a hyperpolarization of the mitochondrial membrane potential which could, like erlotinib, also lead to increased ROS production^41, 42^. In melanoma cells the combination of the BRAF inhibitor vemurafenib with the K_Ca_3.1 blocker TRAM-34 resulted in the release of proapoptotic, mitochondrial factors^16^. As far as the interpretation of our own results is concerned the reduced cell number @ t = 72 h can of course be a consequence of both effects: induction of apoptosis or inhibition of the cell cycle. In melanoma cells the authors noted a depolarization of the mitochondrial membrane potential instead of the anticipated hyperpolarization. In our view this is likely a secondary effect. The combined application of vemurafenib and TRAM-34 could have caused a ROS-induced secondary opening of the permeability transition pore in the inner mitochondrial membrane and thereby caused the mitochondrial depolarization^41^. Finally, K_Ca_3.1 channels can also indirectly affect EGFR-dependent proliferation by providing the electrochemical driving force for TRPC1-mediated Ca^2+^ entry^43^.

Cell migration critically depends on cytoskeletal dynamics^44^ as well as on tightly regulated ion and water fluxes^45^. EGFR activation promotes migration in multiple ways (reviewed in^46^). For example, in oesophageal cancer erlotinib inhibits cell migration by impairing Rho GTPase or focal adhesion kinase (FAK) activity^47^. K_Ca_1.1 channels are known to interact with FAK^48^. By controlling (local) volume homeostasis K_Ca_3.1 and K_Ca_1.1 channels indirectly also affect actin dynamics in migrating cells^49, 50^.

Because of their synergistic and complementary effects it is a meaningful therapeutic strategy to combine EGFR inhibitors such as erlotinib or related compounds and K_Ca_ channel blockers such as senicapoc or paxilline. If the results of our in vitro experiments are applicable in an in vivo setting, one of the advantages will be a dose reduction of the drugs. Our results show that the drug combination inhibits migration and proliferation already at low concentrations that are ineffective when given as “monotherapy”. Dose reduction of erlotinib is a relevant issue since there is ample evidence that the frequency and severity of its side effects correlate with the plasma concentration^51^. It is not yet known whether this would be critical for senicapoc as well. So far, only a few hundred patients with sickle cell anemia have been treated with senicapoc. No major adverse effects had been observed in this patient cohort^52^.

## Supplementary Information

Supplementary Information.
