# Supplementary figures and images for: KCa channel blockers increase effectiveness of the EGF receptor TK inhibitor erlotinib in non-small cell lung cancer cells (A549)
Source: Sci Rep. 2021 Sep 15;11:18330. doi: 10.1038/s41598-021-97406-0 (PMC8443639; doi:10.1038/s41598-021-97406-0)

**Fig. 1Ba-suppl.**

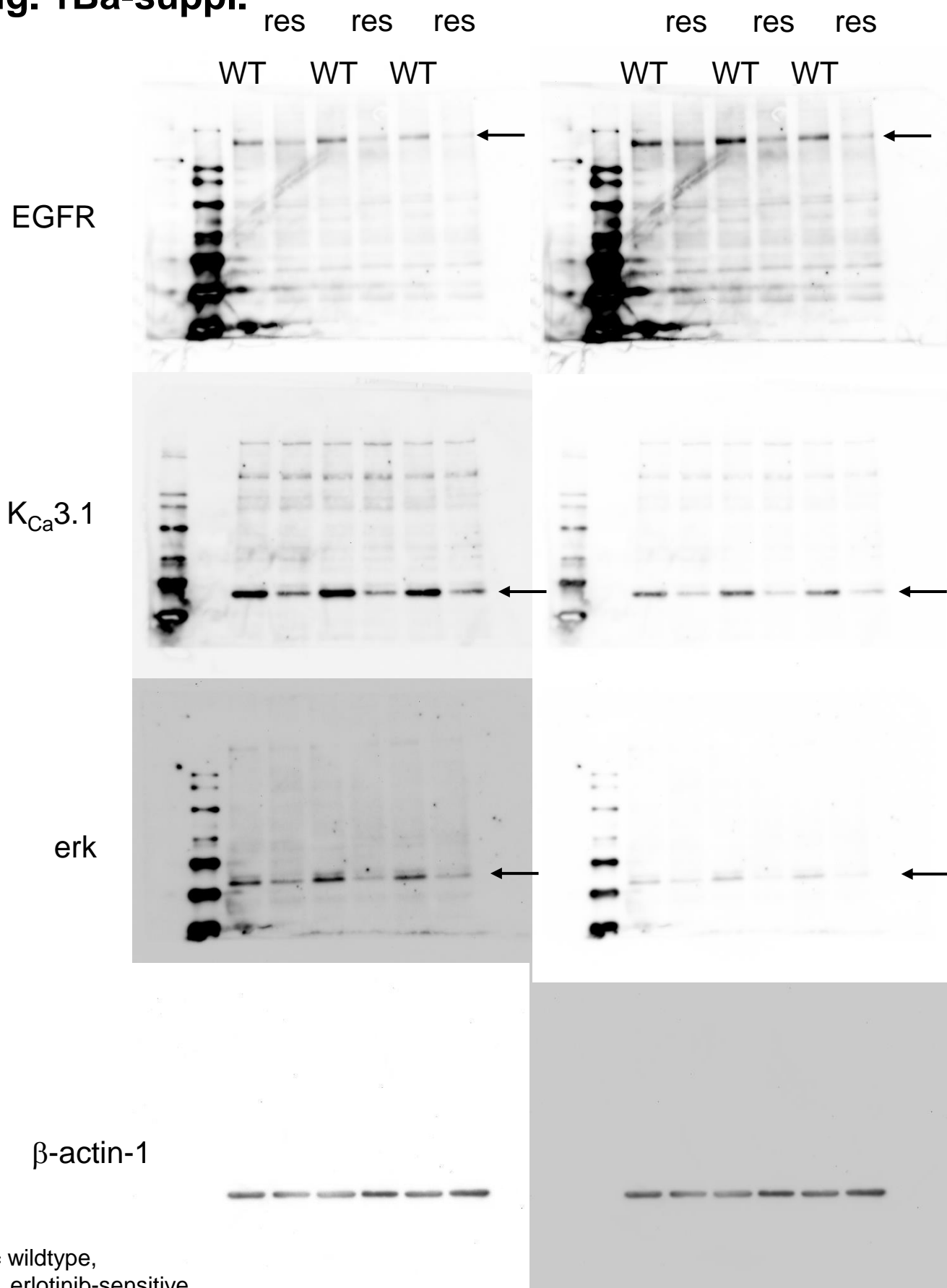

**Fig. 1Ba-suppl.**

$\beta$ -actin-2

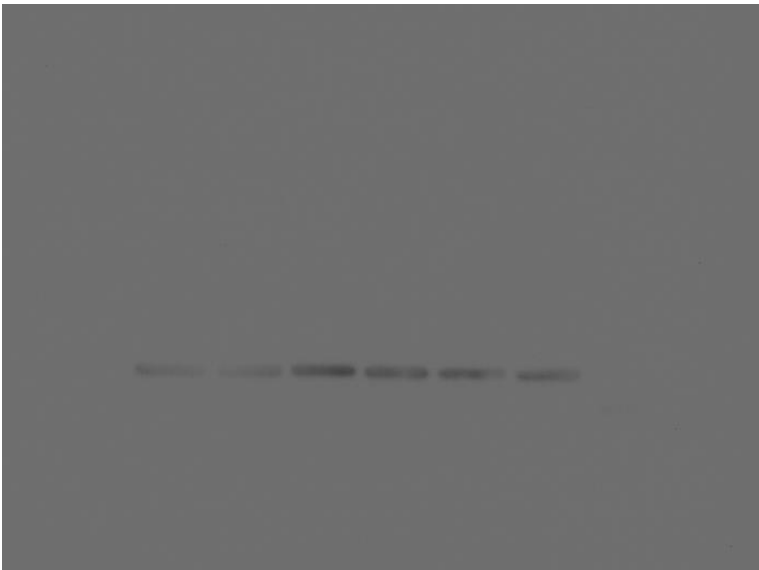

**Fig. 1Bb-suppl.**

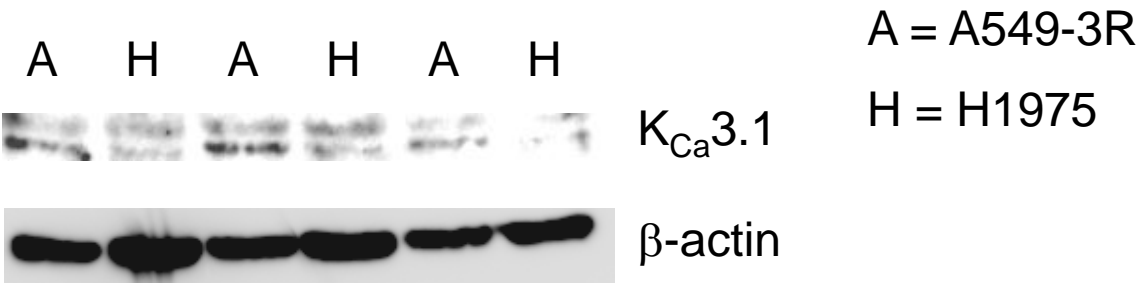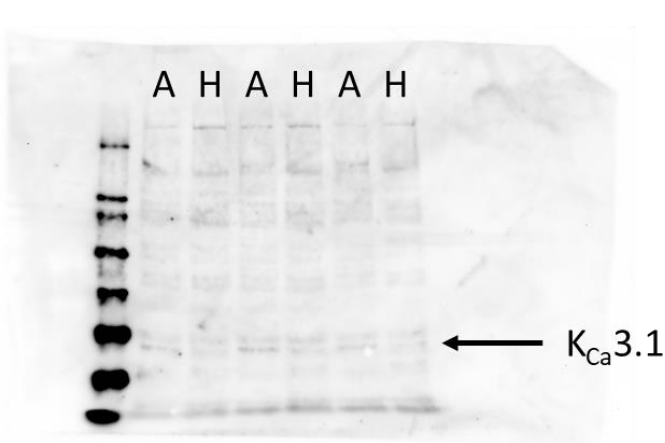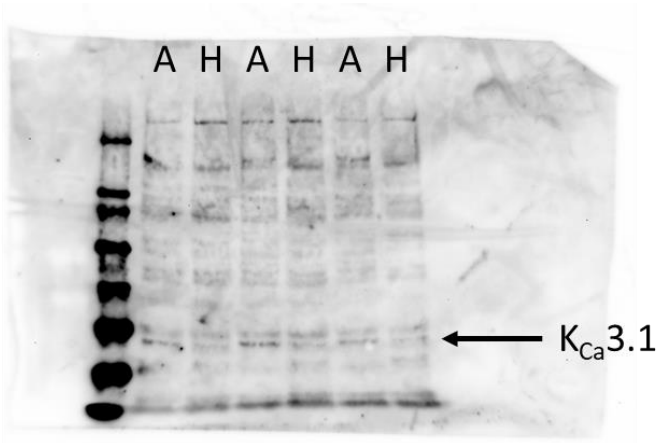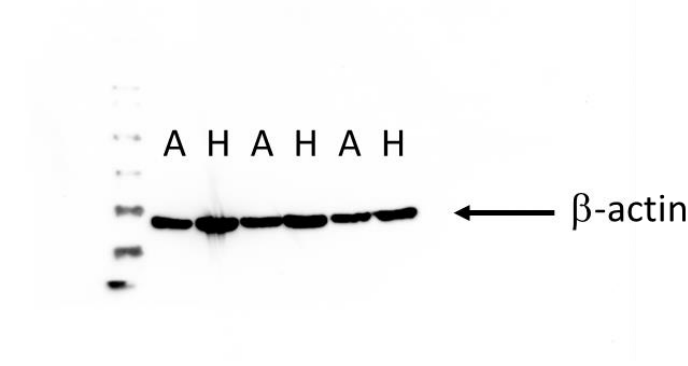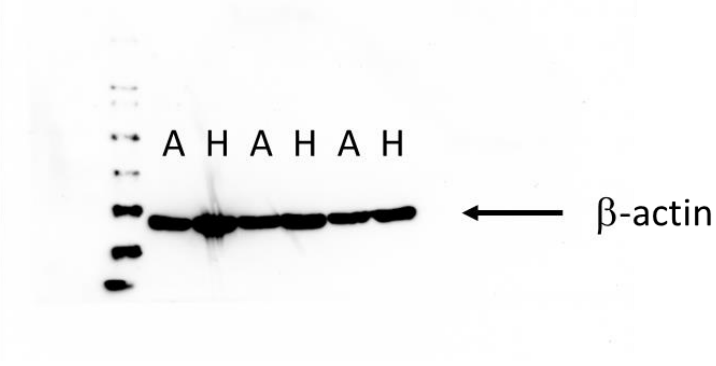

**Fig. 7A-suppl.**

n = normoxia h = hypoxia

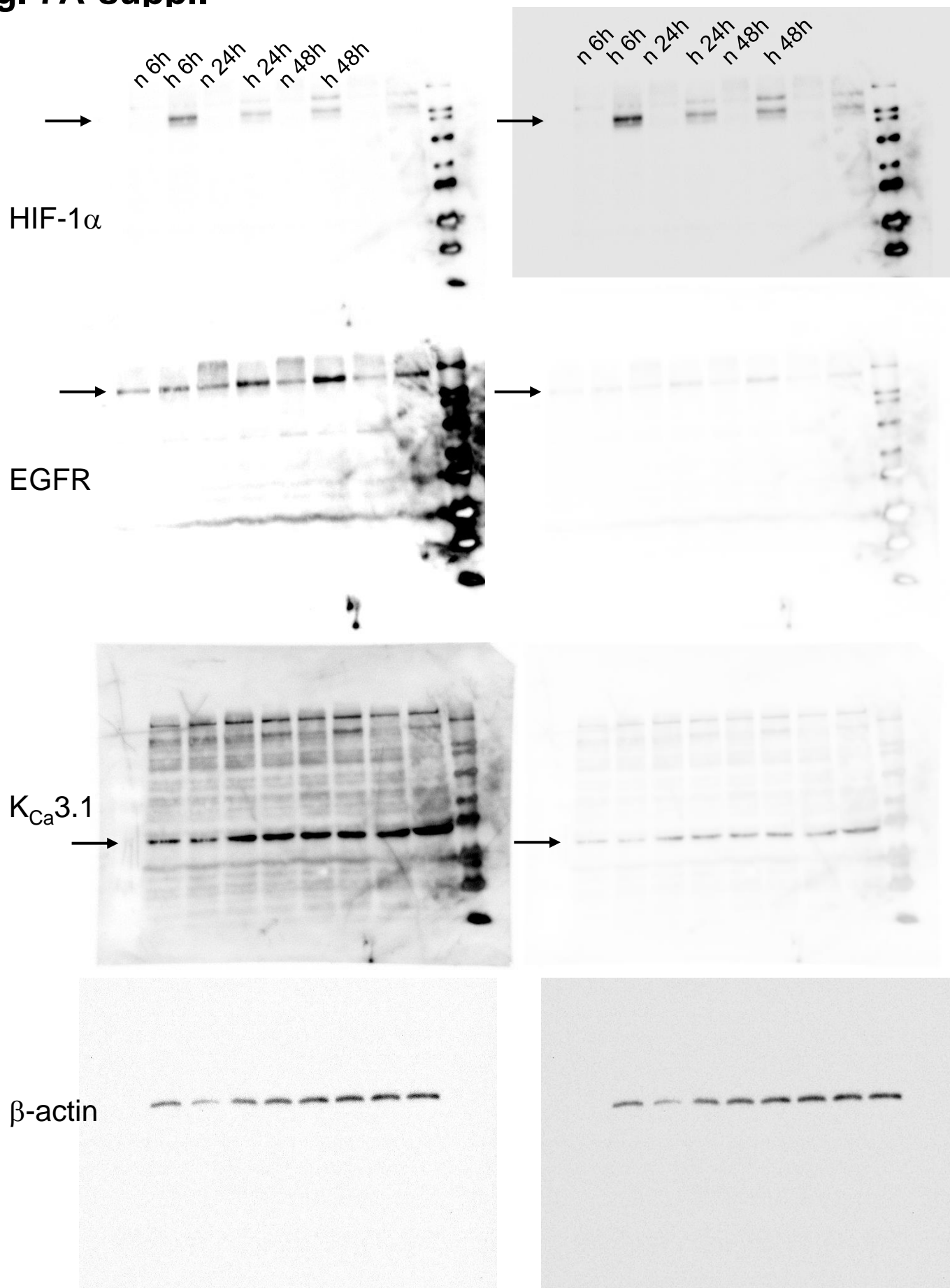

Supplement: Supplementary file 1 — Supplementary Information. [file 41598_2021_97406_MOESM1_ESM.pdf]
